# Anatomy and Surgical Implications of the Lumbar Epidural Venous Plexus: A Risk Zone Framework

**DOI:** 10.7759/cureus.113878

**Published:** 2026-08-03

**Authors:** Stylianos Kapetanakis, Georgia Antoniadou, Paschalis Tsioulas, Christos Siopis, Mikail Chatzivasiliadis

**Affiliations:** 1 Spine Department and Deformities, Interbalkan European Medical Center, Thessaloniki, GRC; 2 2nd Department of Orthopaedics, General Hospital of Thessaloniki "G. Gennimatas", Thessaloniki, GRC

**Keywords:** endoscopic and microscopic spine surgery, epidural veins, intraoperative bleeding, lumbar spine, lumbar venous plexus, vertebral venous plexus

## Abstract

The lumbar epidural venous plexus (LEVP) is a predominantly valveless venous network within the epidural space and an important source of intraoperative bleeding during lumbar spine surgery. Although its anatomy is well described, its practical relationship to surgical exposure and bleeding behavior remains underemphasized.

This narrative review synthesizes the anatomical organization, communications, spatial relationships, and variability of the LEVP and translates these findings into a procedure-oriented framework of venous bleeding risk zones. Based on the relationship of the plexus to the posterior longitudinal ligament (PLL), dura mater, nerve roots, lateral recess, and intervertebral foramen, four clinically relevant zones are proposed: the ventral epidural, lateral recess/axillary, foraminal, and posterior epidural zones. Each zone is associated with distinct surgical exposures, characteristic bleeding patterns, technical pitfalls, and hemostatic considerations. This framework emphasizes that epidural venous bleeding is not random but follows reproducible, anatomy-driven patterns. Recognizing the LEVP as an organized and surgically relevant system may improve anatomical anticipation, intraoperative decision-making, and procedural safety during lumbar spine surgery.

## Introduction and background

The lumbar epidural venous plexus (LEVP) is a complex, valveless venous network within the epidural space of the spinal canal [1,2]. It represents a regional component of the internal vertebral venous system and forms part of the broader interconnected network commonly referred to as Batson’s plexus [3,4]. Within the lumbar spine, this plexus is typically organized into anterior and posterior components, located along the posterior vertebral bodies and within the dorsal epidural space adjacent to the laminae [3].

Although its anatomical organization has been well described, the LEVP remains a frequent and underappreciated source of intraoperative bleeding [5]. This is particularly relevant in minimally invasive and endoscopic spine surgery, where the restricted working corridor and reliance on optical visualization render even low-volume venous bleeding clinically significant [5,6]. In this setting, venous bleeding can rapidly obscure the surgical field, impair anatomical orientation, prolong operative time, and increase the risk of iatrogenic neural injury [7,8].

Despite a substantial body of anatomical literature, there remains a lack of procedure-oriented frameworks that translate venous anatomy into practical surgical guidance [9]. In particular, surgeons lack a structured understanding of where bleeding is most likely to occur, which venous structures are encountered during specific procedural steps, and how these patterns influence operative difficulty.

Unlike previous anatomical reviews, this manuscript proposes a clinically oriented bleeding-risk classification based on surgical anatomy and procedural exposure. By linking anatomical patterns to surgical corridors and procedural steps, it aims to provide a practical reference to improve intraoperative anticipation and management of epidural venous bleeding.

## Review

Narrative review methodology

This narrative review was developed through a focused search of PubMed and the reference lists of relevant articles. Search terms included combinations related to the LEVP, internal vertebral venous plexus, epidural veins, vertebral venous anatomy, lumbar epidural space, nerve-root venous anatomy, endoscopic spine surgery, intraoperative bleeding, and hemostasis. English-language anatomical, imaging, clinical, and surgical studies were considered when they directly addressed LEVP anatomy, anatomical variation, surgical exposure, bleeding behavior, or venous management. Titles and abstracts were reviewed for relevance, followed by full-text assessment of potentially eligible articles. Evidence was synthesized narratively by organizing findings according to anatomical region, variation, surgical exposure, and characteristic bleeding pattern. Because this was not a systematic review, no formal protocol, exhaustive screening process, or risk-of-bias assessment was undertaken, which limits reproducibility and should be considered a limitation.

Anatomy of the LEVP

Overview and Definition

The vertebral venous system constitutes a continuous, longitudinally oriented network responsible for venous drainage of the spinal column and surrounding structures, traditionally described under the umbrella of Batson’s plexus [10]. Contemporary anatomical studies distinguish this system into internal and external vertebral venous plexuses, which communicate extensively through basivertebral and intervertebral veins, forming an integrated cranio-caudal venous pathway [1,2,9]. Within this framework, the LEVP represents the lumbar segment of the internal vertebral venous plexus, situated within the epidural space and characterized by a dense, anastomotic architecture composed of interconnected longitudinal channels [1,11].

Classically, the vertebral venous system has been described as entirely valveless, permitting bidirectional blood flow and facilitating communication between major venous territories [12]. However, this concept has been challenged by Stringer et al., who demonstrated through cadaveric microdissection that valves are present in a proportion of the external vertebral venous plexus, while the internal plexus maintains a predominantly valveless configuration [2]. This distinction highlights that the functional properties of the vertebral venous system are compartment-specific rather than uniform.

A critical refinement of vertebral venous anatomy was provided by Groen et al., who used intravenous polymer injection techniques to demonstrate that the internal vertebral venous plexus is not composed of discrete vessels but instead forms a highly interconnected three-dimensional network with consistent anterior dominance and extensive transverse communications [13]. These findings have become the anatomical foundation for understanding the organization of the LEVP as a continuous, plexiform system rather than a series of isolated veins.

Core Architecture

Within the lumbar region, the internal vertebral venous plexus is arranged as a system of four principal longitudinal channels, consisting of paired anterior and posterior components that extend along the length of the spinal canal [2]. The anterior internal plexus represents the dominant and most consistent structure, forming large longitudinal venous trunks that course along the posterior aspect of the vertebral bodies [2]. Groen et al. demonstrated that these trunks exhibit a characteristic segmental pattern, diverging laterally at the level of the intervertebral discs and reconverging toward the midline over the vertebral bodies through retrocorporeal transverse channels, thereby integrating with basivertebral venous drainage [3,4]. This configuration results in a structurally organized anterior network with predictable topography across lumbar levels. This anterior-posterior asymmetry was further reinforced by Demondion et al., whose MRI-anatomic study of the lumbar venous plexuses demonstrated a regular paired anterior arrangement with consistent midline communication, supporting the concept that the LEVP represents an organized longitudinal system with reproducible anterior architecture rather than a random epidural venous meshwork [14].

**Figure 1:**
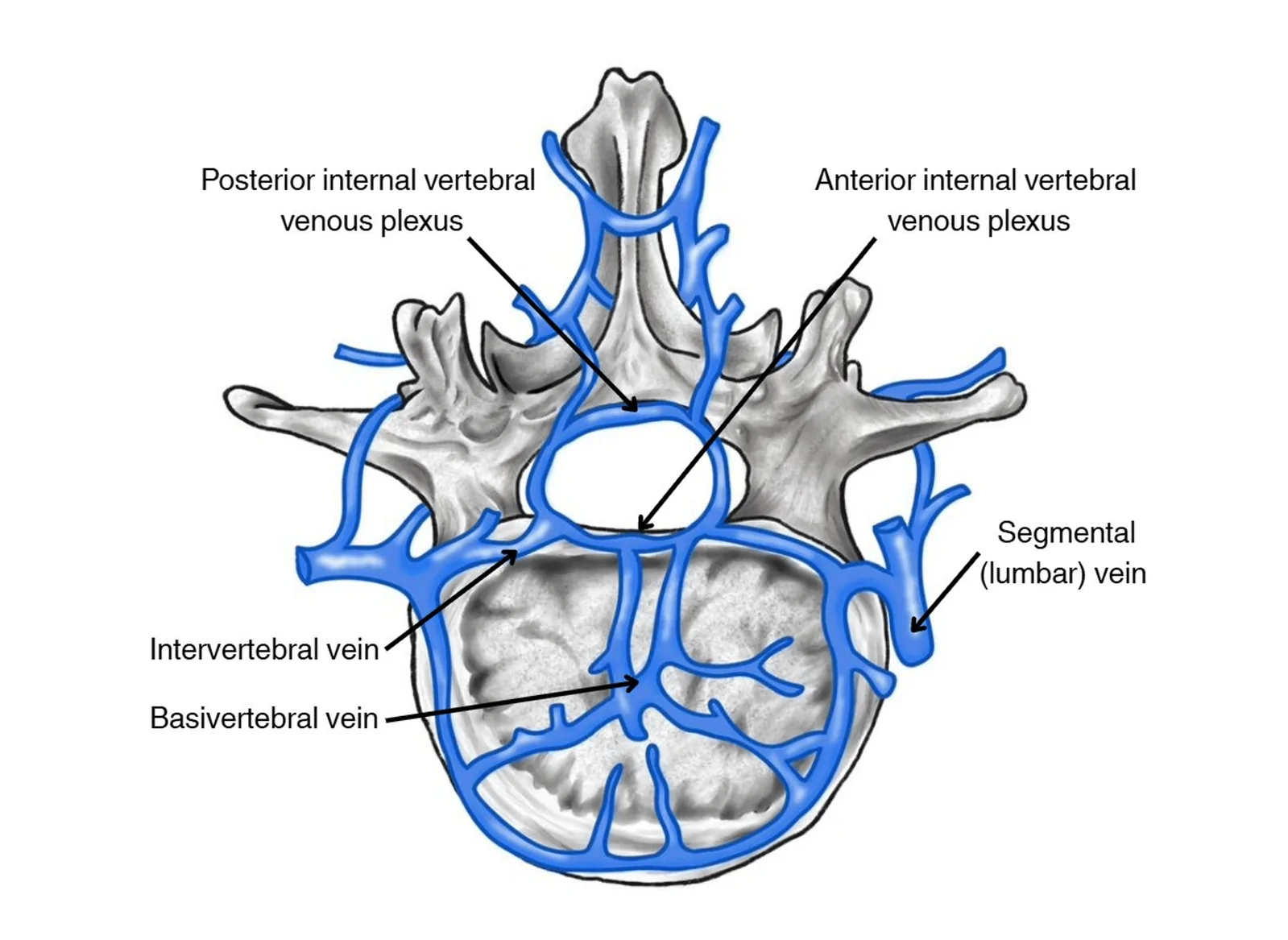
Axial representation of the LEVP and its principal communications. This figure is an original author-created schematic using Procreate version 5.3.15 (Savage Interactive Pty Ltd, Hobart, Tasmania, Australia) and was not generated using AI. LEVP: lumbar epidural venous plexus

In contrast, the posterior internal plexus demonstrates greater variability in both morphology and distribution. Chaynes et al. described this component as consisting of thinner longitudinal channels interconnected by transverse bridging veins, often forming a "ladder-like" configuration beneath the vertebral arches [15]. In the lumbar spine, this posterior network may become more complex, with broader transverse connections and occasional accessory paramedian longitudinal vessels reported in anatomical studies [3,15]. Communication between the anterior and posterior systems is achieved through circumferential anastomoses, particularly at the mid-vertebral level, where ring-like venous connections unify the longitudinal channels into a continuous architectural framework [3]. The organization of the anterior and posterior internal vertebral venous plexuses, together with their basivertebral and segmental communications, is illustrated in Figure 1.

Communications

The LEVP maintains extensive communication with adjacent venous systems through a series of intraosseous and foraminal pathways that integrate it into a continuous vertebral drainage network [16]. Basivertebral veins form a primary conduit between the vertebral body and the anterior internal plexus, traversing the cancellous bone to drain intraosseous blood directly into the epidural venous system [3,4]. At each spinal level, the internal plexus further communicates with the external vertebral venous system through intervertebral (foraminal) veins, which accompany the exiting nerve roots and connect the epidural network to the paravertebral and segmental venous systems [9,17].

These foraminal pathways form a consistent route for venous outflow, linking the epidural space to systemic circulation and facilitating multidirectional flow across compartments. Groen et al. emphasized that these segmental connections contribute to the continuity of the internal vertebral venous plexus, allowing redistribution of blood across adjacent levels rather than confinement to a single segment [3]. Collectively, these intraosseous and intervertebral communications establish the LEVP as a highly interconnected and low-resistance network, capable of rapid redistribution of venous flow across both longitudinal and segmental axes.

Anatomic relationships

Posterior Longitudinal Ligament (PLL) and Anterior Epidural Space

Within the lumbar spine, the anterior component of the internal vertebral venous plexus occupies the anterior epidural space along the posterior aspect of the vertebral bodies and demonstrates a consistent spatial relationship with the PLL [18]. Detailed anatomical investigations have shown that the anterior epidural veins are not freely suspended but instead lie in close apposition to the deep layer of the PLL, which functions as a midline partition separating paired venous channels into right and left compartments [2,19,20]. These longitudinal venous trunks are typically positioned lateral to the ligament within shallow recesses formed between the PLL and the posterior vertebral body, contributing to a reproducible ventral epidural configuration rather than a diffuse venous distribution.

Segmental transverse venous connections course across the posterior vertebral body, linking the paired anterior channels and integrating the plexus with intraosseous drainage pathways via the basivertebral system [3,4,21]. Imaging-based anatomical studies have further supported this organized ventral arrangement, demonstrating that the anterior epidural venous plexus follows a predictable pattern along the posterior vertebral body with consistent midline communication [16,22]. Collectively, these findings indicate that the anterior epidural venous plexus is structurally organized in direct association with the PLL and posterior vertebral body, forming a defined anatomical corridor rather than an irregular venous network.

Dura Mater and Thecal Sac

The LEVP lies within the epidural compartment immediately external to the dura mater, embedded within a fibro-fatty environment rather than suspended in an empty space [19,23]. Parkin and Harrison demonstrated that the lumbar epidural space contains a posterior deposit of epidural fat together with the internal vertebral venous plexus, emphasizing that the dural sac is surrounded by a structured compartment of veins, connective tissue, and fat rather than a simple potential cavity [19]. Wiltse further described the lumbar extradural space as being organized by the peridural membrane and circumneural sheath, which helps explain why the venous plexus maintains a close and mechanically supported relationship to the thecal sac rather than existing as a freely mobile venous layer [24].

This spatial relationship is further reinforced by the surrounding connective-tissue framework. Santos and Kalhorn showed that posterolateral epidural ligaments arise from the dura dorsal to the nerve root sleeves and blend with the peridural membrane, which supports the concept that the epidural veins occupy a compartment stabilized by fibrous attachments in continuity with the dural envelope [25]. In parallel, Uchikado et al. emphasized that the dural sac is enveloped by fibrous connective tissue and adjacent epidural fat in the lumbar canal, with the epidural venous plexus developing within this soft-tissue environment rather than as an isolated vascular structure [26]. Collectively, these observations indicate that the LEVP is circumdurally apposed within a fibrofatty epidural scaffold, which contributes to the confined nature of the epidural space once this compartment is disrupted.

Nerve Roots, Lateral Recess, and Foraminal Transition

At each lumbar level, the internal vertebral venous plexus extends laterally from the central canal toward the exiting nerve roots, where it assumes a distinct perineural configuration. The nerve root is enclosed within a dural sleeve that is intimately surrounded by a network of epidural veins, forming a circumneural venous envelope rather than an isolated vascular structure [16,24]. As this venous network courses laterally through the lateral recess, it becomes increasingly concentrated around the root-pedicle interface, reflecting the transition from the central epidural compartment to the foraminal space [27].

Within the axillary region of the exiting nerve root, this venous plexus is embedded in epidural fat and connective tissue, often rendering individual vessels difficult to distinguish intraoperatively [3]. Endoscopic anatomical studies have emphasized that this axillary fat conceals a dense venous network, which may only become apparent once the tissue is mobilized or disrupted [26]. This region therefore represents a transition zone where veins are not only anatomically concentrated but also visually obscured.

As the plexus continues into the intervertebral foramen, the perineural venous network converges to form the foraminal veins, which establish the primary connection between the internal vertebral venous plexus and the external paravertebral venous system [16,28]. These veins accompany the exiting nerve root and maintain close spatial proximity along its course, reinforcing that the foramen is not solely a neural passage but a complex neurovascular corridor. Collectively, this arrangement supports the concept that the lateral recess and intervertebral foramen function as a continuous neurovascular transition zone, where the LEVP is intimately integrated with the nerve root and surrounding soft-tissue structures.

The principal spatial relationships and organizational features of the LEVP are summarized in Table 1 [2,3,16,18,19,24,26].

**Table 1:** Principal anatomic relationships of the LEVP. Source: [2,3,16,18,19,24,26] LEVP: lumbar epidural venous plexus; PLL: posterior longitudinal ligament

Anatomic region	Spatial relationship to LEVP	Organization/key features
PLL/anterior epidural space	Ventral plexus runs along the posterior vertebral body, in close apposition to the deep layer of the PLL	Paired longitudinal venous channels separated by the PLL; interconnected by transverse veins; direct communication with basivertebral venous system
Dura mater/thecal sac	Plexus lies immediately external to the dura, within the epidural space and surrounding the thecal sac	Embedded in epidural fat; supported by peridural membrane and connective tissue; forms a structured fibrofatty compartment rather than a free space
Lateral recess/foraminal transition	Plexus extends laterally from the central canal around the nerve root and into the intervertebral foramen	Perineural venous envelope surrounding the root; increased venous concentration at the root-pedicle interface; partially concealed within epidural fat and connective tissue

Variations

Inter-individual Morphologic Variability

Although the LEVP is commonly described as a four-channel longitudinal system, its morphology varies substantially between individuals and even between adjacent regions of the same specimen. In the classic Araldite injection study by Groen et al., the internal vertebral venous plexus did not appear as a fixed arrangement of simple veins, but rather as a continuum ranging from relatively flattened, discrete longitudinal trunks to more irregular plexiform configurations with multiple parallel channels and focal dilatations [13]. This variability was particularly evident in the anterior component, where the venous lumen was frequently subdivided by trabeculations that ranged from delicate membranous partitions to thicker bridging structures, creating a multilayered channel system rather than a single uninterrupted conduit [13]. By contrast, the posterior plexus appeared less trabeculated and showed greater segmental and inter-individual irregularity, a pattern also supported by the microsurgical observations of Chaynes et al. and the broader structural reassessment by Stringer et al. [2,13,15].

In practical terms, some specimens demonstrate smoother and more channelized venous trunks, whereas others show a more reticular or sinusoidal appearance, especially within the anterior epidural compartment. Developmental data also support the idea that this variability is intrinsic to the system: Groen et al. found that, in fetuses at 21-25 weeks’ gestation, the anterior plexus already resembles the adult pattern more closely than the posterior plexus, while transverse interconnections remain comparatively underdeveloped, indicating that part of the adult variation likely reflects differential maturation of the venous network [3]. Together, these findings support the view that inter-individual variation in the LEVP is not incidental, but a fundamental feature of its morphology.

Level-Specific Variations

The morphology of the LEVP demonstrates clear variation along the cranio-caudal axis, with progressive enlargement and increasing structural complexity toward the lower lumbar spine. Radiologic and applied anatomical studies have shown that the epidural space itself increases in depth and width caudally, reaching approximately 5-6 mm in the lumbar region compared to significantly smaller dimensions at cervical and thoracic levels, thereby accommodating a more voluminous venous network [29]. Within this expanded compartment, the anterior epidural venous plexus exhibits its greatest development at the L4-L5 levels, where longitudinal venous channels may demonstrate increased caliber and a more plexiform, interconnected configuration compared to more cranial levels [2,3].

Imaging-based analyses further support this regional specialization, demonstrating that the lumbar epidural space contains a denser vascular and fibro-fatty environment compared to more cranial segments, with epidural fat distribution and venous structures varying predictably across levels [30]. In particular, the posterior epidural space is relatively wider and enriched with fat, whereas the anterior compartment, which is bounded by the PLL and vertebral bodies, contains the dominant venous plexus [30]. At lower lumbar levels, this combination of increased epidural space volume and concentrated venous architecture results in a crowded ventral epidural environment, especially adjacent to the L4-L5 disc.

Collectively, these findings indicate that the LEVP is not uniformly distributed but instead demonstrates level-dependent structural adaptation, with maximal venous development in the lower lumbar spine.

Degenerative and Pathologic Remodeling

In degenerative lumbar disease, the morphology of the epidural venous plexus may be altered by both mechanical compression and secondary venous congestion. Imaging and surgical series have shown that epidural venous dilatation or varicosity is not merely an incidental finding, but may accompany lumbar canal stenosis and contribute to root compression within an already narrowed canal [31,32]. In a surgical series of symptomatic lumbar stenosis, Ju et al. reported that epidural venous varicosity was observed in most patients and most commonly involved patterns that directly compressed adjacent neural structures [31]. Similarly, Slin’ko and Al-Qashqish described lumbar epidural varices as acquired lesions related to dilatation of the internal vertebral venous plexus, often occurring in association with disc degeneration and segmental instability rather than as isolated vascular anomalies [33].

These observations suggest that degenerative remodeling of the lumbar spine may influence the LEVP in at least two ways: first, by reducing available epidural space through disc collapse, facet arthropathy, and ligamentous hypertrophy; and second, by promoting local venous engorgement and distortion of the normal plexus architecture. More recent imaging work has also shown that dilatation of the spinal epidural venous plexus correlates with adjacent epidural fat and may become radiologically appreciable in symptomatic patients, further supporting the view that venous morphology is responsive to local structural and hemodynamic conditions rather than anatomically fixed [34,35]. Taken together, these findings indicate that variation of the LEVP is not purely developmental or inter-individual, but may also reflect secondary remodeling in the setting of degenerative lumbar pathology.

The principal patterns of variation in the LEVP are summarized in Table 2 [2,3,13,15,29-35].

**Table 2:** Principal patterns of variation in the LEVP. Source: [2,3,13,15,29-35] LEVP: lumbar epidural venous plexus

Variation domain	Main pattern	Representative features
Overall morphologic pattern	The plexus ranges from relatively simple longitudinal channels to more complex, plexiform networks	Some specimens show smooth, well-defined venous trunks, while others demonstrate multiple interconnected channels with focal dilatations and irregular, reticular configurations
Anterior-posterior asymmetry	The anterior plexus is generally more developed and structurally organized than the posterior component	The anterior plexus often appears trabeculated and multilayered, whereas the posterior plexus is thinner, less organized, and shows greater segmental variability
Level-dependent variation	Venous structures increase in size and complexity toward the lower lumbar spine	The epidural space becomes wider caudally, allowing for a more prominent and densely interconnected venous network, particularly at L4-L5
Degenerative/pathologic remodeling	Venous architecture may become distorted in the presence of degenerative lumbar disease	Dilated or varicose epidural veins, crowding within stenotic segments, and altered relationships with adjacent neural structures may be observed

Risk zones and implications

The proposed risk-zone classification is conceptual and derived from synthesis of the available anatomical and surgical literature. Despite extensive anatomical characterization, its intraoperative behavior remains underappreciated, and epidural bleeding is often approached as diffuse and difficult to control.

The LEVP is a thin-walled, highly anastomotic, predominantly valveless network extending across the anterior, lateral, foraminal, and posterior epidural compartments, closely related to the PLL, dural sac, and nerve root sleeves. As a result, bleeding typically arises from interconnected venous channels rather than a single source, producing low-pressure yet spatially extensive bleeding that rapidly compromises visualization. This happens particularly in minimally invasive and endoscopic surgery [5].

On this basis, a procedure-oriented framework can be made by dividing the LEVP into distinct risk zones based on regional anatomy and surgical exposure. This framework is predictive rather than descriptive: each zone is encountered at a specific operative step and produces a characteristic bleeding pattern, requiring a zone-specific hemostatic strategy. These patterns are further influenced by systemic and positional factors, particularly in the prone position, where impaired venous return may exacerbate epidural venous engorgement [36].

The principal anatomical features, surgical exposures, characteristic bleeding patterns, and management principles for each proposed venous risk zone are summarized in Table 3 [5,15,16,26,29-32,36].

**Table 3:** Anatomy-based venous risk zones of the LEVP. Source: [5,15,16,26,29-32,36] LEVP: lumbar epidural venous plexus; PLL: posterior longitudinal ligament; DRG: dorsal root ganglion

Risk zone	Typical surgical exposure	Dominant venous anatomy	Characteristic bleeding pattern	Main pitfall & guiding principle
Ventral epidural zone	Disc work, PLL detachment, posterior vertebral body exposure	Anterior longitudinal epidural channels with retrocorporeal/basivertebral communications	Diffuse, non-pulsatile, broad-surface bleeding	Pitfall: chasing a single bleeding point. Principle: treat as network bleeding; use tamponade, controlled irrigation, staged exposure, and avoid unnecessary PLL stripping.
Lateral recess/axillary zone	Laminotomy, medial facetectomy, traversing root decompression	Perineural venous plexus at the traversing root and root–pedicle interface	Localized but abrupt bleeding during exposure	Pitfall: blind coagulation after sudden “unmasking.” Principle: proceed with layer-by-layer exposure and identify the venous plexus before coagulation.
Foraminal zone	Transforaminal access, exiting-root mobilization, foraminal decompression	Foraminal veins and perineural venous envelope around the exiting root/DRG	Focal, persistent bleeding in a narrow corridor	Pitfall: prioritizing complete hemostasis over neural safety. Principle: use low-energy, precise hemostasis; avoid excessive compression or coagulation near the DRG.
Posterior epidural zone	Interlaminar exposure, dorsal epidural decompression	Posterior internal vertebral venous plexus with thinner longitudinal channels and transverse connections	Low-volume venous oozing	Pitfall: underestimating slow oozing or relying only on irrigation. Principle: maintain a clear field early; recognize that hydrotamponade may mask persistent bleeding.

Ventral Epidural Zone

The ventral epidural zone corresponds to the anterior compartment of the internal vertebral venous plexus, located along the posterior surface of the vertebral bodies and closely related to the PLL. In this region, paired longitudinal venous channels course on either side of the PLL and are interconnected by transverse retrocorporeal veins that receive direct input from the basivertebral system, forming a continuous interface between intraosseous and epidural venous drainage [15,37]. This is important surgically because bleeding in this zone is rarely caused by one isolated vessel. Rather than representing discrete vessels, the anterior plexus forms a dense, longitudinally organized but highly interconnected network embedded within the ventral epidural compartment, which is the most dominant venous component of the lumbar epidural space [37,38].

The structural organization of this region explains its clinical behavior. The anterior plexus has multiple anastomotic channels and communicates with segmental and intraosseous venous pathways, allowing blood flow to redistribute across adjacent levels and compartments [16]. Because this system is predominantly valveless, fluctuations in intra-abdominal and intrathoracic pressure may be transmitted into the epidural veins, causing variable engorgement, especially in the prone position or when caval return is impaired [16,36]. Therefore, the main mistake in this zone is to treat the bleeding as a focal problem. When the anterior plexus is disrupted, bleeding often arises from several connected channels across a broad surface, not from a single point that can simply be coagulated.

From a surgical perspective, this zone is encountered during procedures involving the posterior vertebral body, intervertebral disc, or PLL, including discectomy, endplate preparation, and PLL detachment. Once violated, the anterior plexus typically produces diffuse, non-pulsatile bleeding that is difficult to localize and control. Chasing the bleeding point with repeated coagulation may enlarge the exposed venous surface, worsen visualization, and increase the risk of thermal or mechanical injury to nearby neural structures. For this reason, management should be based on the nature of the bleeding: broad-surface tamponade, controlled irrigation, patience, and staged exposure are usually more logical than aggressive focal coagulation. Excessive or premature detachment of the PLL may further disrupt this network and exacerbate bleeding, emphasizing the importance of controlled, incremental exposure in this region. In practical terms, the ventral zone should be approached as a network-bleeding zone: the goal is not always to find “the” bleeding vessel but to reduce venous filling, limit further exposure, and regain visualization without creating a larger bleeding surface.

Lateral Recess and Axillary Zone

The lateral recess and nerve root axilla represent a transition zone in which the internal vertebral venous plexus reorganizes into a perineural configuration closely associated with the traversing nerve root. Within this region, epidural veins extend laterally from the central canal and envelop the dural root sleeve, forming a circumneural venous network embedded within epidural fat and contained within the peridural membrane rather than existing as isolated vessels [39]. This creates a compact neurovascular environment at the root-pedicle interface, where longitudinal epidural drainage transitions into segmental outflow.

An important feature of this zone is that the venous network is partially concealed under normal anatomical conditions. The lateral recess is bounded by the lamina, facet joint, and ligamentum flavum, which obscure the underlying venous structures until surgical exposure occurs [27]. As a result, bleeding in this region is often not encountered gradually, but appears suddenly at the moment of decompression. During laminotomy or medial facetectomy, removal of the ligamentum flavum and bony overhang may abruptly expose the perineural venous plexus, leading to what can be described as an “unmasking” phenomenon [27]. A common misconception is that this bleeding is unexpected or abnormal, while in reality it reflects normal anatomy that was previously hidden. This is supported by operative and imaging observations showing that epidural veins in the lateral canal are frequently not appreciated preoperatively and only become evident once disrupted [32,40].

Beyond its surgical relevance, the perineural venous plexus in this region also has clinical significance. Pathologic dilation of these veins may compress the nerve root and produce radicular symptoms, mimicking disc herniation or spinal stenosis [32]. Epidural venous varices have been reported in up to 0.1%-4.5% of lumbar spine surgeries and may be associated with both neural compression and significant intraoperative bleeding [41]. This reinforces that the venous structures in this zone are not incidental, but functionally relevant and, in some cases, directly symptomatic.

From a surgical standpoint, bleeding in this zone differs fundamentally from the ventral compartment. Rather than diffuse network bleeding, the lateral recess typically produces focal but strategically critical bleeding adjacent to the traversing nerve root. This zone is encountered during laminotomy, medial facetectomy, and lateral canal decompression, where stepwise exposure reveals the perineural venous plexus. The key difficulty is not the volume of bleeding, but its location. Hemostasis must be achieved in a confined space directly adjacent to neural structures.

The main surgical mistake in this region is aggressive or blind coagulation immediately after exposure. Because these veins are embedded in fat and connective tissue, they are often poorly visualized when first encountered. Attempting to control bleeding without clear identification of the structure may lead to unnecessary nerve root manipulation or thermal injury. In contrast to the ventral zone, broad tamponade is less effective here. Management should instead focus on controlled, layer-by-layer exposure, early recognition of the venous plexus, and precise, low-energy hemostasis once the anatomy is clearly defined.

Consequently, the lateral recess functions as a “hidden” venous risk zone, where the problem is not vessel size, but the timing of exposure and proximity to neural elements. Bleeding becomes problematic at the exact moment visualization is lost during decompression, making this the most unpredictable phase rather than the most severe bleeding scenario.

Foraminal Zone

The foraminal zone represents a neurovascular transition corridor where the internal vertebral venous plexus communicates with the external paravertebral venous system through the foraminal veins. Borg et al. emphasized that each nerve root is enclosed by a dural sleeve surrounded by a venous plexus, which converges within the foramen to form the foraminal vein before joining the segmental and paraspinal venous system [16]. As a result, the foramen is not simply a neural passage, but a tightly packed space in which the exiting nerve root, dorsal root ganglion (DRG), epidural fat, and venous structures coexist in direct contact. Uchikado et al. similarly showed that the epidural venous plexus extends into the foramen and may be closely adherent to the dural sleeve and DRG, particularly in endoscopic views [26].

From a surgical standpoint, bleeding in this zone is usually produced by traction, mobilization, or decompression of the exiting nerve root and its surrounding venous envelope rather than by injury to a single dominant vessel. This zone is encountered during transforaminal approaches, including transforaminal endoscopic lumbar discectomy (TELD), foraminoplasty, and foraminal decompression, where surgical manipulation occurs directly within the root-venous complex. Because the foraminal veins form a bridging system between the internal and external venous plexuses, disruption may lead to localized but persistent bleeding with continuous inflow, even when the initial source appears limited [16]. This creates a situation where even small amounts of bleeding are difficult to clear, as both the working space and irrigation outflow are restricted.

Unlike the ventral compartment, where bleeding is diffuse, foraminal bleeding is typically focal but disproportionately difficult to control due to its location. Hemostasis must be achieved in a narrow corridor directly adjacent to the exiting nerve root and DRG. The main surgical mistake in this zone is prioritizing bleeding control over neural safety. Aggressive bipolar coagulation, repeated instrument contact, or excessive compression may control bleeding, but at the cost of DRG irritation. This is clinically relevant, as even minor trauma to the DRG may result in significant postoperative dysesthesia or persistent radicular pain.

For this reason, the goal in the foraminal zone is not to complete elimination of bleeding at all costs, but controlled management while preserving neural integrity. Low-energy coagulation, minimal manipulation, and precise instrument handling are preferred over aggressive attempts at complete hemostasis. In some cases, accepting limited residual oozing is safer than repeated attempts at full control in this confined space.

Taken together, the foraminal zone represents the most technically constrained and neurologically sensitive venous risk zone. Here, the limiting factor is not the severity of bleeding, but the inability to control it without risking neural injury. This distinguishes it from the other zones and underscores the need for a different surgical mindset during transforaminal and foraminal procedures.

Posterior Epidural Zone

The posterior epidural zone contains the posterior internal vertebral venous plexus, located within the dorsal epidural compartment anterior to the laminae and ligamentum flavum. This plexus is typically less developed and more variable than the anterior component [2,15,29]. Morphologically, it consists of thinner longitudinal channels connected by transverse bridging veins in a ladder-like arrangement, although its caliber and pattern vary between levels and between individuals [2,15]. In contrast to the ventral plexus, the posterior venous system is more fragmented and less dominant, which explains its different bleeding behavior.

Because the posterior epidural space in the lumbar spine is relatively wider and contains abundant epidural fat, bleeding in this zone is usually less forceful than in the ventral compartment. However, the main issue in this region is not the amount of bleeding, but its effect on visualization. Even low-volume venous oozing can rapidly contaminate the operative field, particularly in confined posterior or endoscopic corridors [29,30].

This is especially relevant in minimally invasive and endoscopic procedures. Irrigation may temporarily control bleeding through hydrotamponade, creating the impression that hemostasis has been achieved, while persistent low-flow bleeding continues in the background. Once irrigation is reduced, the field may quickly deteriorate again [31,42]. This can lead to a cycle of repeated clearing of the field without addressing the underlying source.

The main surgical mistake in this zone is underestimating this type of bleeding. Because it is low-pressure and non-pulsatile, it is often tolerated initially. However, persistent oozing progressively degrades visualization, prolongs operative time, and may increase the risk of orientation errors, particularly during endoscopic work.

From a surgical standpoint, management differs from both the ventral and foraminal zones. Aggressive coagulation is usually unnecessary, but passive observation is equally problematic. Instead, early and controlled hemostasis of small venous channels, combined with efficient workflow and maintenance of a clear field, is key. Accordingly, the posterior epidural zone should not be viewed as a minor bleeding source, but as a low-volume, high-impact zone, where small amounts of bleeding can disproportionately affect surgical performance. The challenge is not controlling severe bleeding, but preventing gradual loss of visualization during critical steps of the procedure.

## Conclusions

The LEVP is a dynamic and clinically relevant system whose anatomical organization influences intraoperative bleeding patterns. The proposed ventral, lateral recess, foraminal, and posterior risk-zone framework may help surgeons anticipate the location and behavior of venous bleeding, particularly during minimally invasive and endoscopic procedures. This narrative review is limited by the absence of systematic methodology and prospective validation. The proposed framework is based primarily on anatomical and surgical literature, while quantitative assessment of bleeding severity was beyond its scope. Cadaveric, imaging, and intraoperative studies are needed to validate and refine the classification. Recognizing epidural venous bleeding as predictable and anatomy-driven may improve surgical planning, hemostatic decision-making, and procedural safety.
